# Development and validation of a pre-chemotherapy nomogram integrating systemic immune-inflammation index and prognostic nutritional index to predict severe adjuvant toxicity in colorectal cancer

**DOI:** 10.3389/fonc.2026.1799706

**Published:** 2026-05-11

**Authors:** Wenjing Li, Hua Zhang, Jie Wu, Liyan Jin

**Affiliations:** 1 Department of Oncology, Wujin Hospital Affiliated with Jiangsu University, Changzhou, Jiangsu, China; 2 Department of Oncology, Wujin Clinical College of Xuzhou Medical University, Changzhou, Jiangsu, China

**Keywords:** chemotherapy toxicity, colorectal cancer, nomogram, prognostic nutritional index, systemic immune-inflammation index

## Abstract

**Background:**

Severe toxicities frequently complicate adjuvant chemotherapy for stage II/III colorectal cancer (CRC), leading to dose reductions and compromised outcomes. While the Systemic Immune-Inflammation Index (SII) and Prognostic Nutritional Index (PNI) are established prognostic markers, their value in predicting chemotherapy tolerance, reflecting physiological reserve, remains understudied. This study evaluated baseline pre-chemotherapy SII and PNI to construct a validated nomogram for predicting severe chemotherapy-induced toxicity (CIT).

**Methods:**

We retrospectively analyzed 306 patients with stage II/III CRC receiving oxaliplatin-based adjuvant chemotherapy (CAPOX or mFOLFOX6). The primary endpoint was grade 3–4 adverse events. Optimal pre-chemotherapy cut-off values were determined via ROC analysis and the Youden index. Independent predictors from multivariate logistic regression were integrated into a nomogram. Performance was assessed via discrimination (apparent and optimism-corrected AUC via 500 bootstrap resamples), calibration (plots and Brier score), and clinical utility (Decision Curve Analysis, DCA).

**Results:**

Severe CIT occurred in 78 (25.5%) patients. Baseline high SII (adjusted OR = 8.56, P < 0.001) and low PNI (adjusted OR = 4.69, P < 0.001) were independent risk factors. The nomogram (Age, SII, PNI) showed excellent discrimination (apparent AUC: 0.868; optimism-corrected AUC: 0.867) and strong calibration (Brier score: 0.1114). Patients in the high-risk group (probability ≥37.4%) experienced a significantly shorter median therapy duration (4.1 vs. 4.8 months) and a markedly lower oxaliplatin relative dose intensity (median 76.7% vs. 100.0%) compared to the low-risk group (both P < 0.001). DCA confirmed the nomogram offered greater net clinical benefit than single markers or traditional assessments.

**Conclusion:**

Pre-chemotherapy elevated SII and decreased PNI are robust predictors of severe toxicity. The proposed nomogram is a reliable, cost-effective tool for risk stratification, enabling risk-adapted strategies like nutritional prehabilitation, dose adjustment, and prophylactic growth factor support for high-risk CRC patients.

## Introduction

Colorectal cancer (CRC) remains a predominant global health challenge, ranking as the third most commonly diagnosed malignancy and the second leading cause of cancer-related mortality worldwide, with approximately 1.9 million new cases and 935,000 deaths reported in 2020 (1). For patients with stage III and high-risk stage II disease, radical resection followed by adjuvant chemotherapy based on fluoropyrimidines and oxaliplatin (e.g., mFOLFOX6 or CAPOX regimens) is the standard of care, significantly reducing recurrence rates and improving overall survival (2, 3). However, the therapeutic window of these cytotoxic agents is narrow. The administration of adjuvant chemotherapy is frequently hampered by severe treatment-related toxicities, including grade 3–4 neutropenia, thrombocytopenia, gastrointestinal reactions, and peripheral neuropathy (4).

Severe chemotherapy-induced toxicity (CIT) is not merely a transient adverse event; it has profound clinical implications. It often necessitates dose reductions, treatment delays, or premature discontinuation of therapy, which can significantly compromise the relative dose intensity (RDI) and overall therapy duration, and subsequently diminish long-term oncological outcomes (4, 5). Furthermore, severe toxicity significantly impairs patients’ quality of life and increases the economic burden due to unplanned hospitalizations (6). Currently, the determination of chemotherapy dosage relies predominantly on body surface area (BSA). However, BSA-based dosing fails to account for inter-individual variability in pharmacokinetics and drug metabolism, leading to substantial disparities in drug exposure and tolerance (7). While the Eastern Cooperative Oncology Group performance status (ECOG PS) is widely used to assess patient fitness, it is inherently subjective and may not accurately reflect the specific physiological reserve required to metabolize and clear cytotoxic drugs (8). Therefore, there is an urgent need to identify objective, accessible, and cost-effective biomarkers to stratify patients based on their risk of severe CIT, thereby enabling personalized management strategies.

Accumulating evidence suggests that the host’s systemic inflammatory response and nutritional status play pivotal roles in the pharmacokinetics and pharmacodynamics of chemotherapeutic agents (9). Systemic inflammation can suppress the activity of hepatic cytochrome P450 (CYP) enzymes, the primary enzymatic system for drug metabolism, thereby inhibiting drug clearance and increasing systemic exposure to cytotoxic agents (10, 11). Simultaneously, malnutrition, characterized by hypoalbuminemia, results in reduced drug-protein binding; this leads to an increase in the free, active fraction of the drug in the circulation, which directly exacerbates cellular toxicity (12). Consequently, composite immuno-nutritional indices have emerged as promising predictive markers.

Among these indices, the Systemic Immune-Inflammation Index (SII) and the Prognostic Nutritional Index (PNI) have gained considerable attention. The SII comprehensively reflects the balance between the host’s inflammatory response and immune status, while the PNI is a robust indicator of nutritional and immunological reserves. While the prognostic value of SII and PNI regarding survival outcomes (Overall Survival and Disease-Free Survival) has been extensively documented in CRC, their specific utility in predicting chemotherapy-induced safety and tolerance remains understudied (13–16). Furthermore, while existing toxicity screening often requires expensive imaging for quantitative body composition analysis (e.g., muscle mass assessment) or specialized genetic testing (e.g., DPYD), SII and PNI are derived from routine blood tests, making them highly accessible in daily clinical practice (17, 18).

Despite isolated reports on inflammatory markers, a practical, validated tool that integrates baseline immunonutritional status to guide adjuvant therapy safety is currently lacking. Therefore, the primary objective of this retrospective study was to investigate the association between pre-chemotherapy (baseline) SII and PNI levels and the occurrence of grade 3–4 adverse events in patients with stage II/III CRC. Unlike previous studies focusing on survival, we specifically targeted the predictive power of these indices for treatment safety. Based on these findings, we aimed to develop and internally validate a parsimonious nomogram, rigorously adjusted for optimism and collinearity, to identify high-risk patients who might benefit from intensive monitoring, nutritional prehabilitation, or proactive dose modifications.

## Materials and methods

### Study design and patient population

This was a retrospective cohort study conducted at Wujin People’s Hospital. We reviewed the medical records of patients with colorectal cancer (CRC) who underwent radical resection between January 2018 and December 2024. The median interval between surgery and the initiation of adjuvant chemotherapy was 27.66 days (range: 14–44 days).

The inclusion criteria were as follows: (1) histologically confirmed colorectal adenocarcinoma; (2) pathological stage II or III according to the 8th edition of the AJCC/UICC TNM staging system; (3) received at least one cycle of standard adjuvant chemotherapy (CAPOX or mFOLFOX6 regimen) after surgery; (4) Eastern Cooperative Oncology Group (ECOG) performance status of 0–1; and (5) available complete medical records and laboratory data. A total of 28 patients were excluded due to incomplete laboratory or clinical records; no data imputation was performed, given the low missingness rate (< 5%) in the initial eligible pool.

The exclusion criteria were: (1) pathological stage I or IV disease; (2) evidence of acute infection, active inflammation, or autoimmune diseases within one week before chemotherapy initiation (to prevent interference with inflammatory markers); (3) previous history of other malignancies or neoadjuvant therapy; (4) incomplete hematological data before the first chemotherapy cycle; and (5) discontinuation of chemotherapy for non-medical reasons.

The study protocol was approved by the Ethics Committee of Wujin People’s Hospital and complied with the Declaration of Helsinki. The requirement for informed consent was waived due to the retrospective nature of the study.

### Data collection and definitions

Clinical and pathological data were retrospectively extracted from electronic medical records, including age, sex, height, weight, primary tumor location, histopathological differentiation grade, TNM stage, comorbidities (defined as the presence of at least one major chronic condition requiring ongoing medical therapy, such as arterial hypertension, diabetes mellitus, or cardiovascular disease), and adjuvant chemotherapy regimen. Body mass index (BMI) was calculated as weight (kg) divided by the square of height (m2).

Peripheral venous blood samples were collected within 7 days before initiation of the first cycle of adjuvant chemotherapy (pre-chemotherapy baseline). To account for potential confounding factors, baseline laboratory values, including serum creatinine (Cr), alanine aminotransferase (ALT), and aspartate aminotransferase (AST), were recorded. Hematological parameters, including absolute neutrophil count (ANC), absolute lymphocyte count (ALC), platelet count (PLT), and serum albumin concentration (Alb), were obtained from routine clinical laboratory reports. Using these parameters, two composite indices were computed:

Systemic Immune-Inflammation Index (SII): SII = (platelet count × neutrophil count)/lymphocyte count
Prognostic Nutritional Index (PNI): PNI = serum albumin (g/L) + 0.005 × total lymphocyte count (per mm^3^)

### Chemotherapy regimens and toxicity assessment

All patients received standard adjuvant chemotherapy regimens per the NCCN Guidelines. Two regimens were administered: CAPOX (Oxaliplatin 130 mg/m^2^ IV on day 1, plus oral capecitabine 1000 mg/m^2^ bid on days 1–14, every 3 weeks; planned for 8 cycles) and mFOLFOX6 (Oxaliplatin 85 mg/m^2^, leucovorin 400 mg/m^2^, and 5-fluorouracil bolus/infusion, every 2 weeks; planned for 12 cycles). Primary granulocyte colony-stimulating factor (G-CSF) prophylaxis (administration before any evidence of neutropenia) was recorded and included as a covariate in the safety analysis.

Treatment exposure was rigorously evaluated using two metrics: overall therapy duration (in months) and the relative dose intensity (RDI) of oxaliplatin. RDI was calculated as the ratio of the actual delivered cumulative dose to the initially prescribed planned cumulative dose based on body surface area (130 mg/m^2^ × 8 for CAPOX, and 85 mg/m^2^ × 12 for mFOLFOX6).

Treatment-related adverse events (AEs) were monitored across all cycles. AE severity was graded according to NCI-CTCAE version 5.0. The primary endpoint was the occurrence of grade ≥3 toxicity during the adjuvant chemotherapy period.

### Statistical analysis

Statistical analyses were conducted using R software (version 4.5.1; The R Foundation for Statistical Computing, Vienna, Austria). Continuous variables were assessed for normality using the Shapiro–Wilk test. Normally distributed variables are presented as mean ± standard deviation (SD) and compared using the independent-samples Student’s t-test; non-normally distributed variables are reported as median [interquartile range, IQR] and compared using the Mann–Whitney U test. Categorical variables are summarized as frequency (percentage) and analyzed using the Chi-square test or Fisher’s exact test, as appropriate. A two-sided P-value < 0.05 was considered statistically significant for all tests.

ROC curve analysis (pROC package) determined optimal pre-chemotherapy cut-off values for SII and PNI via the Youden index. Univariate logistic regression screened candidate predictors; variables with P < 0.10 were considered for the multivariate model. To ensure model parsimony and clinical utility, a stepwise selection process was employed. Multicollinearity among predictors was assessed using the Variance Inflation Factor (VIF), with VIF < 5 considered acceptable.

A nomogram was constructed based on the identified independent predictors using the rms package, with its performance rigorously evaluated across several dimensions. Model discrimination was quantified by the area under the ROC curve (AUC), and to address potential overfitting, internal validation was performed using 500 bootstrap resamples to calculate the optimism-corrected AUC (19, 20). Calibration was assessed graphically via calibration curves and the Brier score, while clinical utility was evaluated through decision curve analysis (DCA) and the identification of an optimal clinical decision threshold (21). Finally, sensitivity analyses were conducted to mitigate potential “circular reasoning” bias, arising from the inclusion of neutrophils in the SII, by separately validating the model’s predictive value for non-hematological toxicities and comparing treatment exposure, defined by therapy duration and relative dose intensity, between risk groups to evaluate the clinical impact of predicted toxicity.

## Results

### Patient characteristics and selection

**Figure 1:**
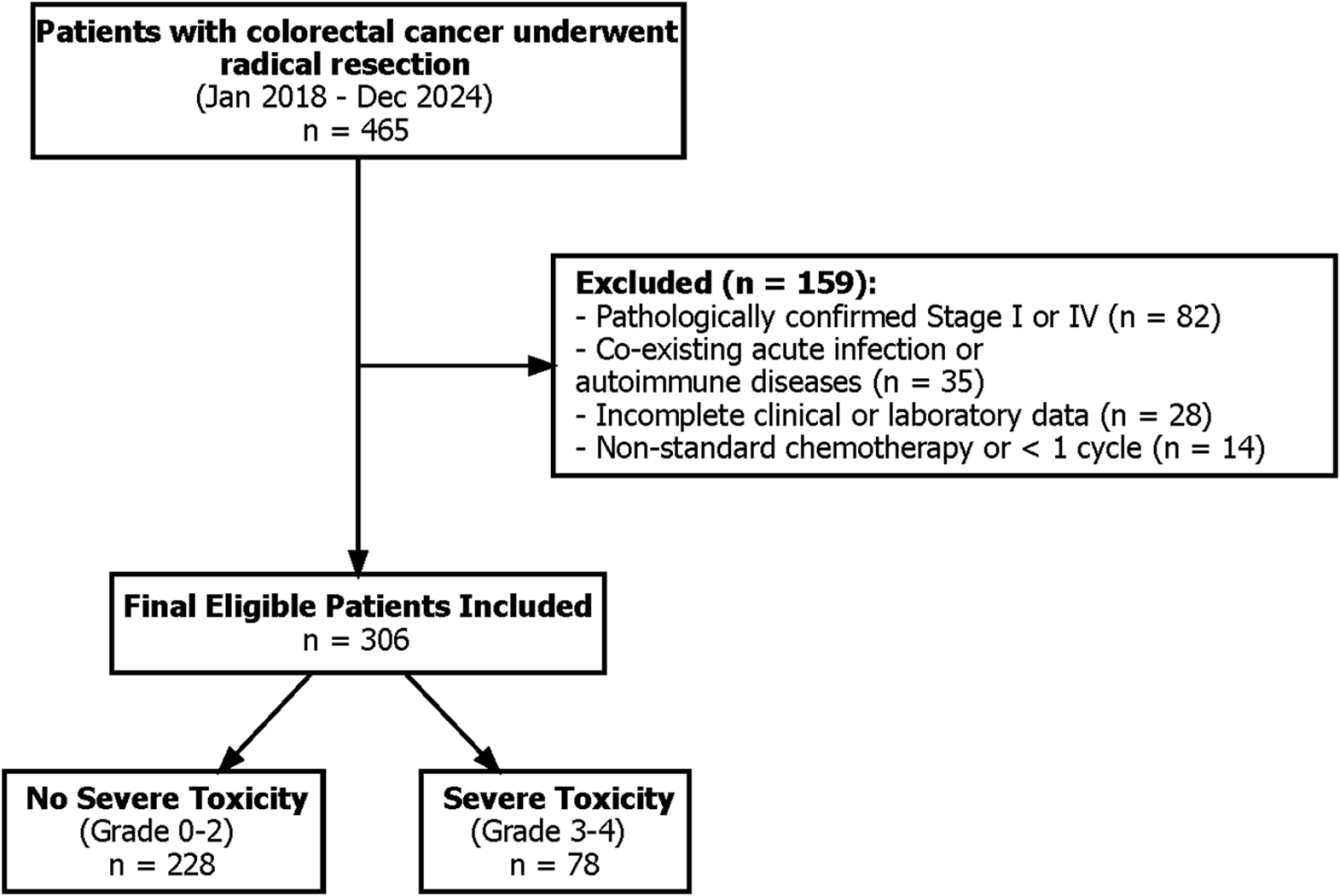
Flowchart of patient selection and study design. A total of 465 patients were initially screened. After excluding patients with pathological stage I/IV disease, evidence of active inflammation, incomplete clinical/hematological records, history of other malignancies, or neoadjuvant therapy, 306 eligible patients were included in the final analysis. Patients were stratified into two cohorts based on the occurrence of severe chemotherapy-induced toxicity (Grade 3–4).

A total of 465 patients with pathologically confirmed stage II or III colorectal cancer who underwent curative-intent radical resection were retrospectively screened between January 2018 and December 2024. After applying prespecified exclusion criteria, 306 patients met all eligibility requirements and were included in the final analysis (**Figure 1**). The median interval between surgical resection and pre-chemotherapy blood collection was 27.7 days (interquartile range [IQR]: 24–31 days), ensuring that inflammatory markers reflected the pre-treatment baseline rather than acute postoperative stress. The median time between blood sample collection and the initiation of the first chemotherapy cycle was 4.0 days (IQR: 2.0–6.0 days).

Within the study cohort, 78 patients (25.5%) developed grade ≥3 chemotherapy-induced toxicity during adjuvant treatment, whereas 228 patients (74.5%) experienced only grade 0–2 toxicity. Baseline clinicopathological characteristics are summarized in **Table 1**. Patients in the severe toxicity group were significantly older (mean age: 64.4 vs. 60.8 years; P = 0.007) and had a lower mean BMI (25.1 vs. 26.0 kg/m^2^; P = 0.001) than those in the non-severe group. Baseline laboratory evaluations revealed that the severe toxicity group had slightly higher mean creatinine levels (88.6 vs. 84.7 μmol/L; P = 0.006), although both remained within clinical normal limits; no significant disparities were observed in baseline liver function (ALT/AST) or the distribution of chemotherapy regimens. Notably, primary G-CSF prophylaxis was utilized in only 4.9% of the total cohort, with no significant difference between groups (P = 0.158). Critically, marked intergroup disparities were observed in systemic inflammatory and nutritional status: the median SII was significantly higher in the severe toxicity group compared to the non-severe group (1184.6 vs. 427.4, P < 0.001), while the median PNI was significantly lower (46.3 vs. 52.9, P < 0.001). No statistically significant differences were detected between groups for sex, primary tumor location, TNM stage, or comorbidity burden (all P > 0.05).

**Table 1:** Baseline clinical and pathological characteristics of the study population.

Characteristics	Total (N = 306)	Severe toxicity group (n = 78)	Non-severe toxicity group (n = 228)	P-value
Age (years), mean ± SD	61.7 ± 10.3	64.4 ± 9.8	60.8 ± 10.4	0.007
Sex, n (%)				0.819
Male	185 (60.5%)	46 (59.0%)	139 (61.0%)	
Female	121 (39.5%)	32 (41.0%)	89 (39.0%)	
BMI (kg/m²), mean ± SD	25.8 ± 3.4	25.1 ± 3.1	26.0 ± 3.5	0.001
Comorbidity, n (%)				0.063
Yes	118 (38.6%)	37 (47.4%)	81 (35.5%)	
No	188 (61.4%)	41 (52.6%)	147 (64.5%)	
Time from surgery to blood collection (days), median (IQR)	27.7 (24.0-31.0)	27.8 (24.1-31.2)	27.6 (23.9-30.9)	0.852
Chemotherapy regimen, n (%)				0.884
CAPOX	156 (51.0%)	41 (52.6%)	115 (50.4%)	
mFOLFOX6	150 (49.0%)	37 (47.4%)	113 (49.6%)	
Primary G-CSF prophylaxis, n (%)	15 (4.9%)	1 (1.3%)	14 (6.1%)	0.158
Baseline creatinine (μmol/L), mean ± SD	85.7 ± 15.2	88.6 ± 14.9	84.7 ± 15.3	0.006
SII, median (IQR)	543.2 (345.1-890.5)	1184.6 (850.2-1560.4)	427.4 (290.5-610.3)	< 0.001
SII category, n (%)				< 0.001
High (> 761.6)	90 (29.4%)	61 (78.2%)	29 (12.7%)	
Low (≤ 761.6)	216 (70.6%)	17 (21.8%)	199 (87.3%)	
PNI, median (IQR)	50.8 (46.5-55.2)	46.3 (42.1-49.5)	52.9 (49.0-56.8)	< 0.001
PNI category, n (%)				< 0.001
High (≥ 49.8)	216 (70.6%)	13 (16.7%)	203 (89.0%)	
Low (< 49.8)	90 (29.4%)	65 (83.3%)	25 (11.0%)	

Data are presented as mean ± SD, standard deviation, median (IQR, interquartile range), or frequency (percentage). Comorbidity was defined as the presence of at least one major chronic comorbid condition (e.g., arterial hypertension, diabetes mellitus, or cardiovascular disease) requiring ongoing medical therapy.SII, Systemic Immune-Inflammation Index; PNI, Prognostic Nutritional Index; BMI, Body Mass Index; G-CSF, Granulocyte colony-stimulating factor. P-values were calculated using the independent-samples Student’s t-test for normally distributed continuous variables, the Mann-Whitney U test for non-normally distributed variables (including SII and PNI medians), and the Chi-square or Fisher’s exact test for categorical variables.

### Optimal cut-off values for SII and PNI

**Figure 2:**
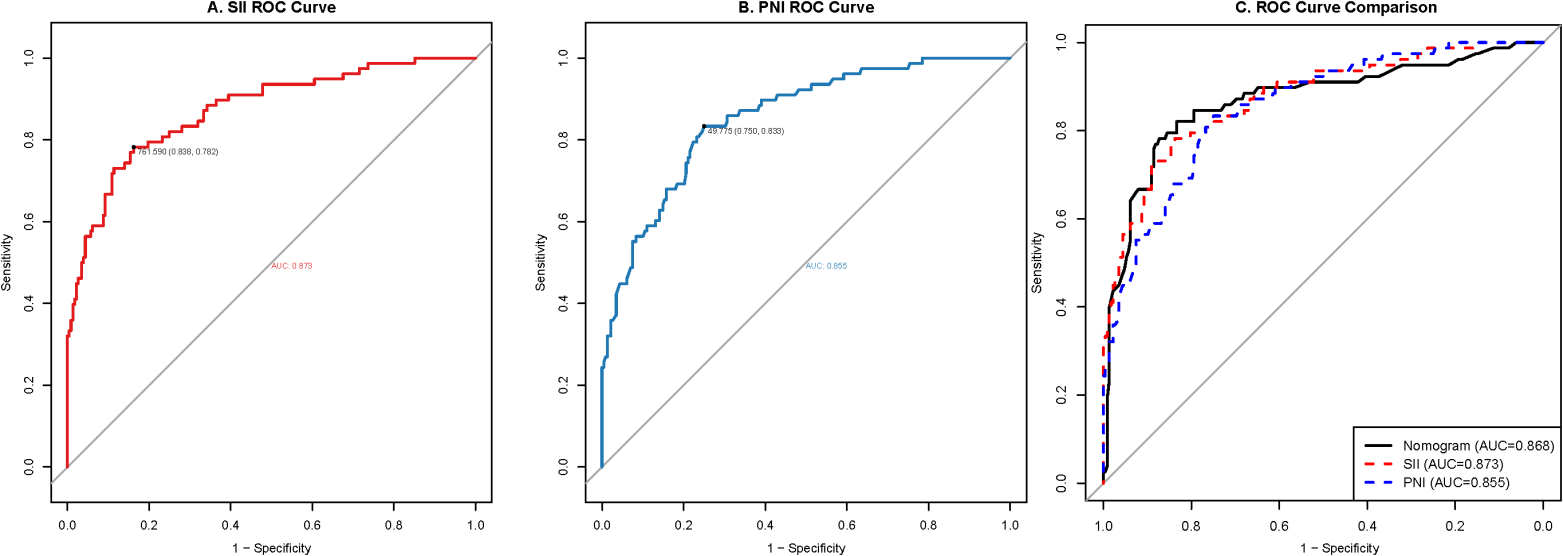
ROC, Receiver operating characteristic curves for pre-chemotherapy inflammatory and nutritional markers. **(A)** Optimal cut-off value for the SII, Systemic Immune-Inflammation Index. **(B)** Optimal cut-off value for the PNI, Prognostic Nutritional Index. **(C)** Comparison of the predictive accuracy (AUC) among the combined nomogram, SII alone, and PNI alone.

ROC curve analysis was performed to determine optimal pre-chemotherapy cut-off values for SII and PNI in predicting grade ≥3 toxicity (**Figure 2**). The optimal SII threshold was 761.6, yielding an AUC of 0.873 (95% CI: 0.838–0.908), sensitivity of 78.2%, and specificity of 83.8% (**Figure 2A**). For PNI, the optimal cut-off was 49.8, with an AUC of 0.855 (95% CI: 0.815–0.895), sensitivity of 83.3%, and specificity of 75.0% (**Figure 2B**). Based on these empirically derived thresholds, patients were dichotomized into high-SII (> 761.6) versus low-SII (≤ 761.6) groups, and high-PNI (≥ 49.8) versus low-PNI (< 49.8) groups for subsequent analyses.

### Univariate and multivariate logistic regression analyses

Univariate logistic regression identified age (P = 0.008), BMI (P = 0.001), baseline creatinine (P = 0.007), cumulative oxaliplatin dose (P < 0.001), SII category (P < 0.001), and PNI category (P < 0.001) as significantly associated with grade ≥3 toxicity (**Table 2**). Comorbidities approached but did not reach statistical significance (P = 0.063). Multicollinearity assessment yielded variance inflation factors (VIF) for all predictors below 2.2 (SII: 2.15; PNI: 2.13), indicating no significant redundancy between the inflammatory and nutritional indices.

**Table 2:** Univariate and multivariate logistic regression analyses for predictors of severe toxicity.

Variables	Univariate analysis		Multivariate analysis	
	OR (95% CI)	P-value	Adjusted OR (95% CI)	P-value
Age (years) *continuous*	1.042 (1.012 - 1.074)	0.008	1.043 (1.009 - 1.079)	0.013
Sex (female vs. male)	1.066 (0.613 - 1.849)	0.819	–	–
BMI (kg/m²) *continuous*	0.825 (0.742 - 0.910)	0.001	–	-*
Tumor location (rectum vs. colon)	0.999 (0.590 - 1.696)	0.999	–	–
TNM stage (III vs. II)	1.258 (0.619 - 2.502)	0.522	–	–
Chemo regimen (mFOLFOX6 vs. CAPOX)	0.963 (0.573 - 1.616)	0.884	–	–
Comorbidity (yes vs. no)	1.644 (0.970 - 2.784)	0.063	–	-*
Baseline creatinine *continuous*	1.037 (1.010 - 1.068)	0.007	–	–
Cumulative oxaliplatin dose	0.998 (0.997 - 0.999)	< 0.001	–	-**
SII category (high vs. low)	10.972 (5.603 - 22.404)	< 0.001	8.561 (3.972 - 18.453)	< 0.001
PNI category (low vs. high)	9.076 (4.801 - 17.653)	< 0.001	4.693 (2.117 - 10.406)	< 0.001

Variables with P < 0.10 in univariate analysis were considered for the multivariate model. *Excluded from the final parsimonious model to prevent overfitting, based on rigorous variable selection and reviewer recommendations. **Cumulative Oxaliplatin Dose is a post-treatment outcome reflecting treatment exposure rather than a pre-chemotherapy baseline predictor, hence it was excluded from the multivariate predictive model. Abbreviations: OR, Odds Ratio; CI, Confidence Interval. Continuous variables (Age, BMI, Baseline Creatinine) were modeled as continuous variables without categorization.

**Figure 3:**
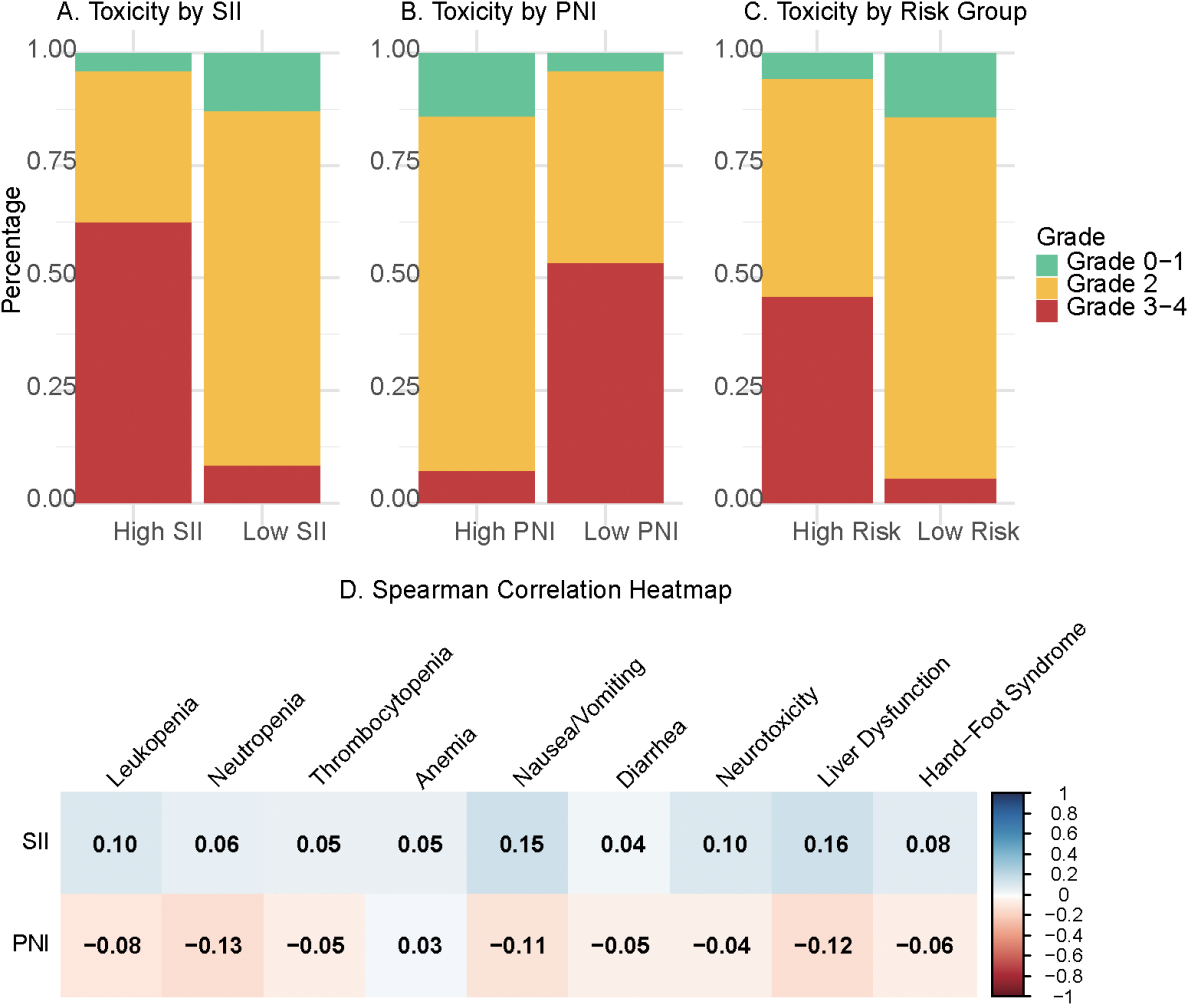
Toxicity distribution and correlation analysis. Incidence of specific grade ≥3 adverse events stratified by **(A)** SII category, **(B)** PNI category, and **(C)** nomogram-derived Risk Group. **(D)** Spearman correlation heatmap illustrating the relationships among baseline SII, PNI, and various specific toxicities.

To ensure a parsimonious and clinically applicable model, multivariate logistic regression confirmed age, SII category, and PNI category as the primary independent predictors. Specifically, patients in the high-SII group exhibited a markedly elevated risk of severe toxicity compared with the low-SII group (adjusted OR = 8.56; 95% CI: 3.97–18.45; P < 0.001). Conversely, patients in the low-PNI group had a substantially higher risk than those in the high-PNI group (adjusted OR = 4.69; 95% CI: 2.12–10.41; P < 0.001). Age remained independently associated with increased risk (adjusted OR = 1.04 per year; 95% CI: 1.01–1.08; P = 0.013). The incidence of grade ≥3 toxicity was significantly higher in the High-Risk Group (75.5% vs. 4.6%; P < 0.001) (**Figure 3**).

### Construction and validation of the nomogram

**Figure 4:**
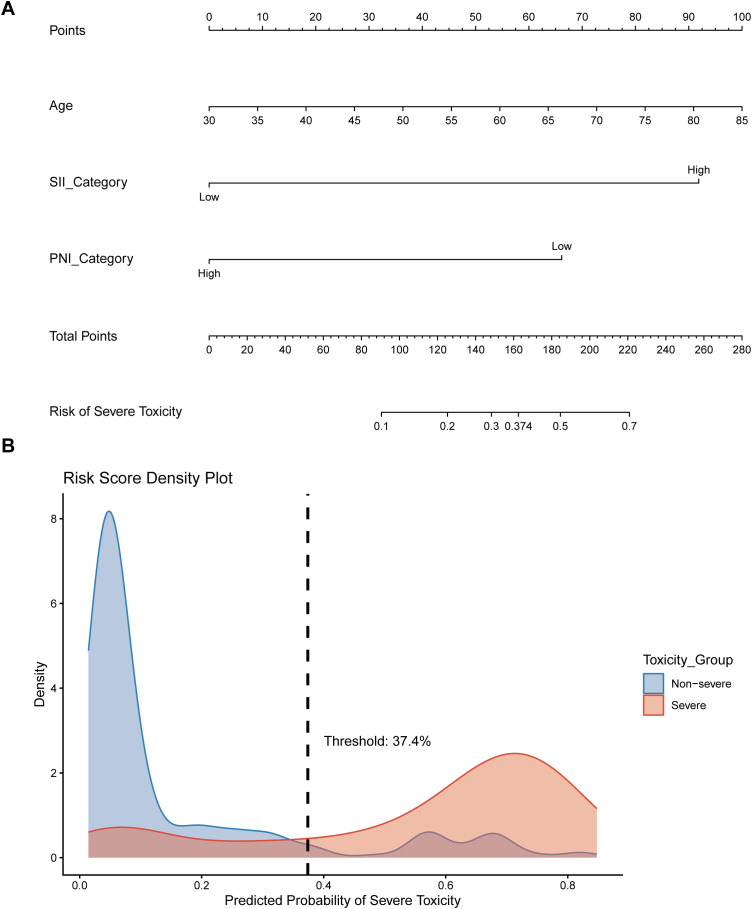
Construction and risk stratification of the predictive nomogram. **(A)** The parsimonious nomogram incorporating age, SII category, and PNI category for predicting grade ≥3 chemotherapy-induced toxicity. **(B)** Risk score density plot illustrating the distribution of predicted probabilities between the severe and non-severe toxicity groups, with the optimal clinical decision threshold identified at 37.4%.

A clinical prediction nomogram was constructed using the three independent predictors identified in the multivariate analysis (age, SII category, and PNI category) (**Figure 4A**). Following reviewer recommendations, BMI and comorbidity count were excluded from the final model to maintain optimal parsimony. In the nomogram, SII category and PNI category contributed the greatest weights to the total risk score. Furthermore, the risk score density plot demonstrated a clear separation between patients with and without severe toxicity (**Figure 4B**).

**Figure 5:**
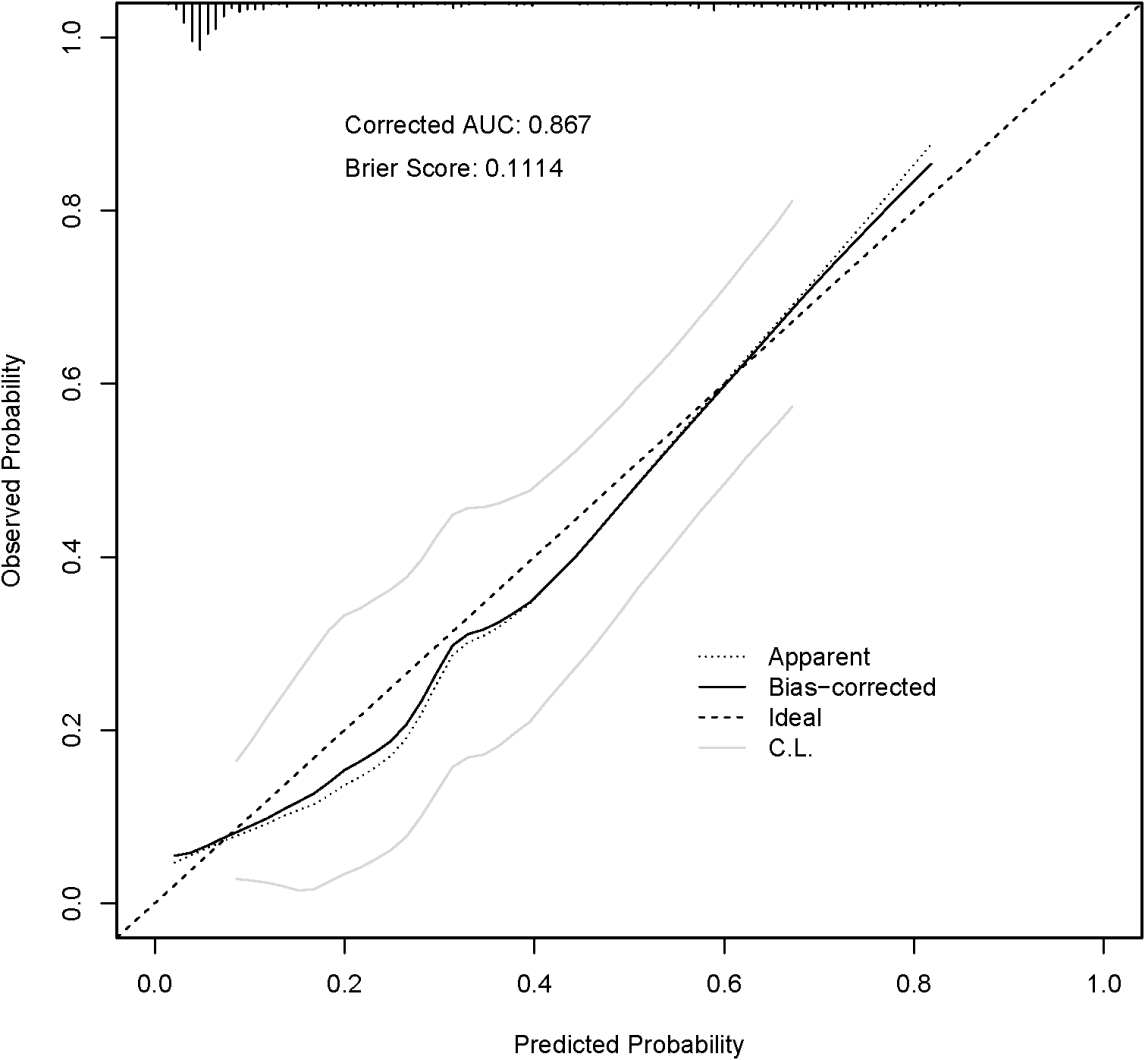
Calibration curve of the nomogram. The x-axis represents the nomogram-predicted probability of severe toxicity, and the y-axis represents the actual observed frequency. Internal validation was performed using 500 bootstrap resamples (bias-corrected AUC = 0.867, Brier score = 0.1114).

The predictive performance of the nomogram was evaluated using ROC analysis and calibration curves. The model demonstrated robust discrimination with an apparent AUC of 0.868. To address potential overfitting, internal validation was performed using 500 bootstrap resamples, yielding an optimism-corrected AUC of 0.867 (**Figure 5**). The Brier score was 0.1114, indicating high predictive accuracy. The calibration curve demonstrated excellent consistency between predicted probabilities and actual observed frequencies. The regression coefficients and intercept for the final model are detailed in **Supplementary Table 1** to facilitate external clinical use.

### Risk stratification and specific toxicity analysis

Using the optimal clinical decision threshold of 37.4% derived from the Youden index, patients were classified into Low-Risk (n=216) and High-Risk (n=90) groups. Furthermore, analysis of treatment exposure revealed that patients in the High-Risk Group experienced a significantly shorter median therapy duration than those in the Low-Risk Group (4.1 vs. 4.8 months; P < 0.001). Additionally, the median Oxaliplatin RDI was markedly lower in the High-Risk Group than in the Low-Risk Group (76.7% vs. 100.0%; P < 0.001; **Supplementary Figure 1**), reflecting a higher rate of premature treatment discontinuation and substantial dose reductions due to severe adverse events.

Subgroup analysis of overall adverse events across the entire cohort (**Table 3**) revealed that neutropenia was the most common severe hematological toxicity (9.5%), while diarrhea (6.2%) and nausea/vomiting (5.2%) were the predominant severe non-hematological toxicities. When stratified by risk (**Table 4**), the High-Risk Group had significantly higher rates of grade 3–4 hematological toxicities, including leukopenia (13.3% vs. 1.4%, P < 0.001), neutropenia (21.1% vs. 4.6%, P < 0.001), and thrombocytopenia (8.9% vs. 0.5%, P < 0.001). Notably, significant differences were also observed for anemia (6.7% vs. 0.5%; P = 0.003) and neurotoxicity (4.4% vs. 0.5%; P = 0.028). Among non-hematological toxicities, the High-Risk Group also exhibited higher rates of severe nausea/vomiting (13.3% vs. 1.9%, P < 0.001), diarrhea (16.7% vs. 1.9%, P < 0.001), liver dysfunction (10.0% vs. 0.5%, P < 0.001), and hand-foot syndrome (6.7% vs. 0.5%, P = 0.003). To mitigate concerns regarding circular reasoning, a sensitivity analysis confirmed that the model remained highly predictive for non-hematological toxicities alone (AUC = 0.826, 95% CI: 0.757–0.895, **Supplementary Figure 2**).

**Table 3:** Overall incidence of severe (grade ≥3) toxicity in the total study cohort.

Toxicity type	No. of patients with grade ≥3 events	Percentage (%)
Hematological toxicities		
Neutropenia	29	9.50%
Leukopenia	15	4.90%
Thrombocytopenia	9	2.90%
Anemia	7	2.30%
Non-hematological toxicities		
Diarrhea	19	6.20%
Nausea/Vomiting	16	5.20%
Liver dysfunction	10	3.30%
Hand-foot syndrome	7	2.30%
Neurotoxicity	5	1.60%
Any grade ≥3 toxicity*	78	25.50%

*Represents the number of distinct patients experiencing at least one grade 3–4 adverse event. The cumulative sum of specific events exceeds the total number of affected patients (n=78) because several patients experienced multiple concurrent overlapping toxicities (e.g., concurrent neutropenia and diarrhea).

**Table 4:** Comparison of specific severe adverse events (grade ≥3) between nomogram-defined risk groups.

Adverse event	Low-risk group (N=216)	High-risk group (N=90)	P-value
Hematological toxicities			
Leukopenia	3 (1.4%)	12 (13.3%)	< 0.001
Neutropenia	10 (4.6%)	19 (21.1%)	< 0.001
Thrombocytopenia	1 (0.5%)	8 (8.9%)	< 0.001
Anemia	1 (0.5%)	6 (6.7%)	0.003
Non-hematological toxicities			
Diarrhea	4 (1.9%)	15 (16.7%)	< 0.001
Nausea & Vomiting	4 (1.9%)	12 (13.3%)	< 0.001
Liver dysfunction	1 (0.5%)	9 (10.0%)	< 0.001
Hand-foot syndrome	1 (0.5%)	6 (6.7%)	0.003
Neurotoxicity	1 (0.5%)	4 (4.4%)	0.028

The study cohort was stratified into Low-Risk and High-Risk groups based on the optimal nomogram-derived probability threshold of 37.4%. Data are presented as frequencies and percentages n (%). P-values were calculated using the Chi-square test or Fisher’s exact test (where expected cell counts were < 5, such as in Thrombocytopenia, Anemia, Liver Dysfunction, Hand-Foot Syndrome, and Neurotoxicity).

### Clinical utility

**Figure 6:**
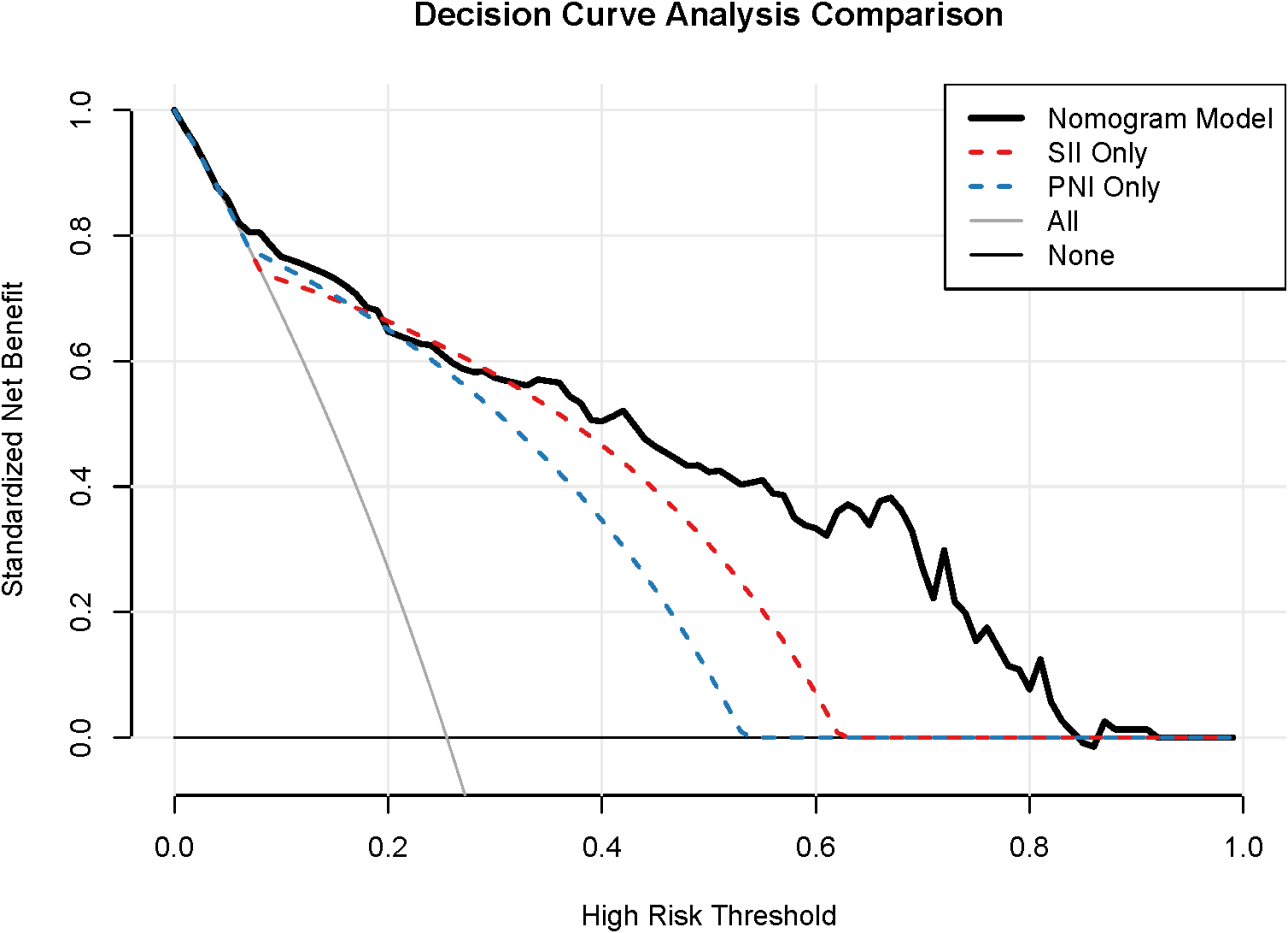
DCA, Decision curve analysis for clinical utility. The DCA demonstrates the standardized net benefit of utilizing the nomogram compared to “treat-all” or “treat-none” strategies across a wide range of high-risk thresholds.

Decision Curve Analysis (DCA) was performed to evaluate the clinical utility of the nomogram (**Figure 6**). The DCA showed that utilizing the nomogram to predict severe toxicity provided a greater net benefit than treating all patients or treating no patients across a wide range of threshold probabilities. Based on the identified 37.4% threshold, the nomogram offers a practical cut-off for initiating prophylactic interventions (e.g., primary G-CSF or intensive nutritional support). The composite model consistently offered superior clinical value compared to single markers.

## Discussion

To the best of our knowledge, this is among the first studies to develop and rigorously validate a baseline composite nomogram integrating the systemic immune-inflammation index (SII) and prognostic nutritional index (PNI) for predicting grade ≥3 chemotherapy-induced toxicity (CIT) specifically in patients with stage II or III colorectal cancer. Our results demonstrate that pre-chemotherapy SII and reduced PNI are independent, biologically plausible predictors of severe adverse events. The final nomogram, derived from a parsimonious model of only three variables (age, SII, and PNI), demonstrated excellent discrimination (apparent AUC = 0.868; optimism-corrected AUC = 0.867) and strong calibration (Brier score = 0.1114). According to the TRIPOD statement (19), internal validation is crucial to assess model reproducibility; the minimal optimism (0.001) observed during 500 bootstrap resamples (20) underscores the model’s stability and reliability for individualized risk stratification.

The biological plausibility underlying the association between systemic inflammation (high SII) and CIT susceptibility is multifactorial. A critical concern often raised is whether SII merely reflects baseline hematological counts that overlap with toxicity definitions. However, our sensitivity analysis confirmed that the model remained highly predictive for non-hematological toxicities alone (AUC = 0.826), suggesting that SII captures a broader state of physiological vulnerability. Furthermore, our study utilized blood samples collected at a median of 27.7 days post-surgery and a median of 4.0 days before chemotherapy initiation, effectively ensuring that the baseline SII reflects chronic systemic inflammation and pre-treatment baseline rather than acute postoperative stress. Systemic inflammation can suppress the activity of hepatic cytochrome P450 (CYP) enzymes, the primary pathway for drug metabolism, thereby inhibiting drug clearance and increasing systemic exposure to cytotoxic agents (10, 11, 18). Pro-inflammatory cytokines like IL-6 directly suppress the transcriptional regulation of these enzymes, heightening the risk of tissue toxicity. Additionally, chronic inflammation (signified by high SII) contributes to a pro-oxidative state and impaired hematopoietic niche function, which may compromise normal tissue resilience against chemotherapy-induced damage (22, 23).

Conversely, the predictive value of PNI highlights the critical role of nutritional reserves in treatment tolerance. Serum albumin, a key component of PNI, serves as a primary drug-binding protein (12). In states of hypoalbuminemia, the fraction of unbound (free) active drug in the circulation increases, exacerbating toxicity even at standard dosages. Although low PNI may share overlapping clinical phenotypes with malnutrition-related conditions like sarcopenia, it was initially developed to assess immunonutritional status, and direct correlations with skeletal muscle mass require further prospective validation (24, 25). Nevertheless, baseline malnutrition and immunological depletion are well-established drivers of dose-limiting toxicities (17). Our multivariate analysis identified low PNI as a potent predictor (adjusted OR = 4.69), reinforcing the concept that nutritional depletion significantly compromises the host’s physiological “buffer” against cytotoxic challenge.

Comparison with previous studies underscores the unique value of our work. While SII and PNI have been extensively documented as prognostic markers for survival (OS/DFS) in various cancers (13–16, 26), their utility as predictive markers for safety has been underexplored. Unlike generic performance scores (e.g., ECOG PS) (8) or dosing based solely on BSA (7), which fail to capture subclinical metabolic deficits, our nomogram provides an objective quantification of biological risk. Despite the inherent inclusion of lymphocyte parameters in both indices, our diagnostic check yielded variance inflation factors (VIF) below 2.2, confirming that SII and PNI provide independent and complementary information without statistical redundancy.

The clinical implications of this work extend beyond risk prediction to enabling evidence-informed, preemptive interventions. Our findings revealed that patients identified as “High-Risk” (risk probability ≥37.4%) experienced a significantly shorter therapy duration (median 4.1 vs. 4.8 months; P < 0.001) and profound reductions in Oxaliplatin relative dose intensity (median 76.7% vs. 100.0%; P < 0.001) compared to the Low-Risk group, emphasizing that severe toxicity is a primary driver of reduced treatment exposure (5). Based on the optimal clinical threshold of 37.4% identified via the Youden index, we propose a risk-adapted framework. Specifically, high-risk patients should receive protocol-driven nutritional prehabilitation before chemotherapy to improve PNI and mitigate catabolic stress (27, 28). Furthermore, primary G-CSF administration should be strongly considered for these individuals, given their 21.1% incidence of grade 3–4 neutropenia, aligning with ASCO guidelines, which recommend prophylaxis for patients with a ≥20% predicted risk (29). Finally, for those with exceptionally high risk scores, a conservative initial dose reduction followed by stepwise escalation may offer a safer alternative to standard-dose induction, potentially reducing the likelihood of unplanned treatment discontinuation and optimizing the balance between therapeutic efficacy and patient safety.

Several limitations warrant acknowledgment. First, the retrospective, single-center design may introduce selection bias, although rigorous exclusion criteria were applied. Importantly, while our overall incidence of grade ≥3 toxicity (25.5%) is slightly lower than that reported in prospective trials like the IDEA collaboration (e.g., 37.6% for FOLFOX), this likely reflects the inherent underreporting of non-hospitalized toxicities (such as moderate nausea or grade 3 peripheral neuropathy) in real-world retrospective records. Second, unmeasured variables, such as germline pharmacogenomic variants (e.g., DPYD) and quantitative body composition metrics, were not incorporated. Third, while internal validation confirmed model stability (20), external validation in prospective, multicenter cohorts is essential to establish generalizability (19). Decision curve analysis (DCA) further confirmed that the nomogram offers superior net benefit compared to single markers across a clinically relevant range of threshold probabilities (21). Furthermore, while our nomogram relies on baseline pre-chemotherapy measurements to facilitate early and proactive risk stratification, it is important to acknowledge that the host’s immune and nutritional status is highly dynamic. Recent literature highlights the significant predictive and prognostic value of longitudinal changes (delta values) in inflammatory and nutritional indices during treatment for both metastatic and resected cancers (30). Future iterations of our model could potentially integrate these dynamic trajectories to provide real-time, adaptive toxicity monitoring throughout the chemotherapy course.

## Conclusion

In conclusion, pre-chemotherapy SII and PNI are powerful, cost-effective predictors of severe adjuvant chemotherapy toxicity in stage II/III colorectal cancer. The proposed nomogram, which integrates routine laboratory parameters and has been rigorously validated for discrimination and calibration, offers a simple tool to transition from “one-size-fits-all” dosing to personalized precision medicine. By identifying patients at high risk of reduced relative dose intensity and premature treatment discontinuation, this model provides a clinical basis for proactive, risk-adapted management. Future prospective trials are warranted to evaluate whether biomarker-guided interventions can reduce toxicity rates and improve treatment completion without compromising oncological outcomes.

## Data Availability

The original contributions presented in the study are included in the article/**Supplementary Material**. Further inquiries can be directed to the corresponding author.

## Supplementary material

Supplementary Table 1 Regression coefficients and intercept of the multivariate logistic regression model.

Supplementary Figure 1 Comparison of treatment exposure between nomogram-defined risk groups. **(A)** Boxplots showing the RDI, relative dose intensity of oxaliplatin, calculated as the ratio of the actual delivered cumulative dose to the initially prescribed planned cumulative dose based on body surface area (8 cycles for CAPOX and 12 cycles for mFOLFOX6). **(B)** Boxplots showing the total therapy duration in months. The High-Risk group demonstrated a significantly lower RDI and a shorter therapy duration compared to the Low-Risk group (both P < 0.001).

Supplementary Figure 2 Sensitivity analysis of the nomogram for predicting severe non-hematological toxicities. ROC curve evaluating the predictive performance of the composite model specifically for non-hematological adverse events (diarrhea, nausea/vomiting, liver dysfunction, hand-foot syndrome, and neurotoxicity), yielding an AUC of 0.826 (95% CI: 0.757–0.895).
