# Supplementary figures and images for: Development and validation of a pre-chemotherapy nomogram integrating systemic immune-inflammation index and prognostic nutritional index to predict severe adjuvant toxicity in colorectal cancer
Source: Front Oncol. 2026 May 11;16:1799706. doi: 10.3389/fonc.2026.1799706 (PMC13198996; doi:10.3389/fonc.2026.1799706)

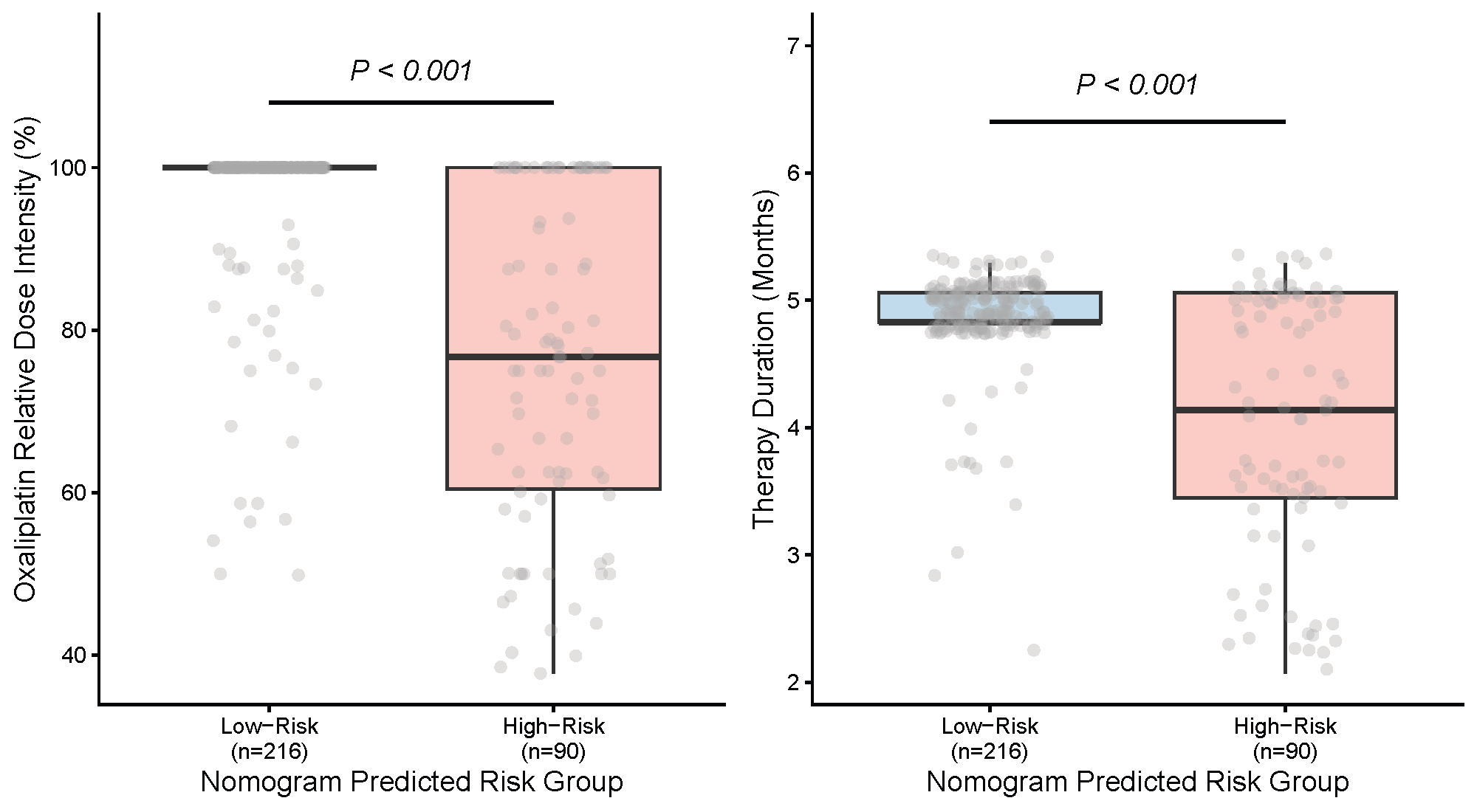

Supplement: Supplementary Figure 1 — Comparison of treatment exposure between nomogram-defined risk groups. (A) Boxplots showing the RDI, relative dose intensity of oxaliplatin, calculated as the ratio of the actual delivered cumulative dose to the initially prescribed planned cumulative dose based on body surface area (8 cycles for CAPOX and 12 cycles for mFOLFOX6). (B) Boxplots showing the total therapy duration in months. The High-Risk group demonstrated a significantly lower RDI and a shorter therapy duration compared to the Low-Risk group (both P < 0.001). [file Image1.tif]

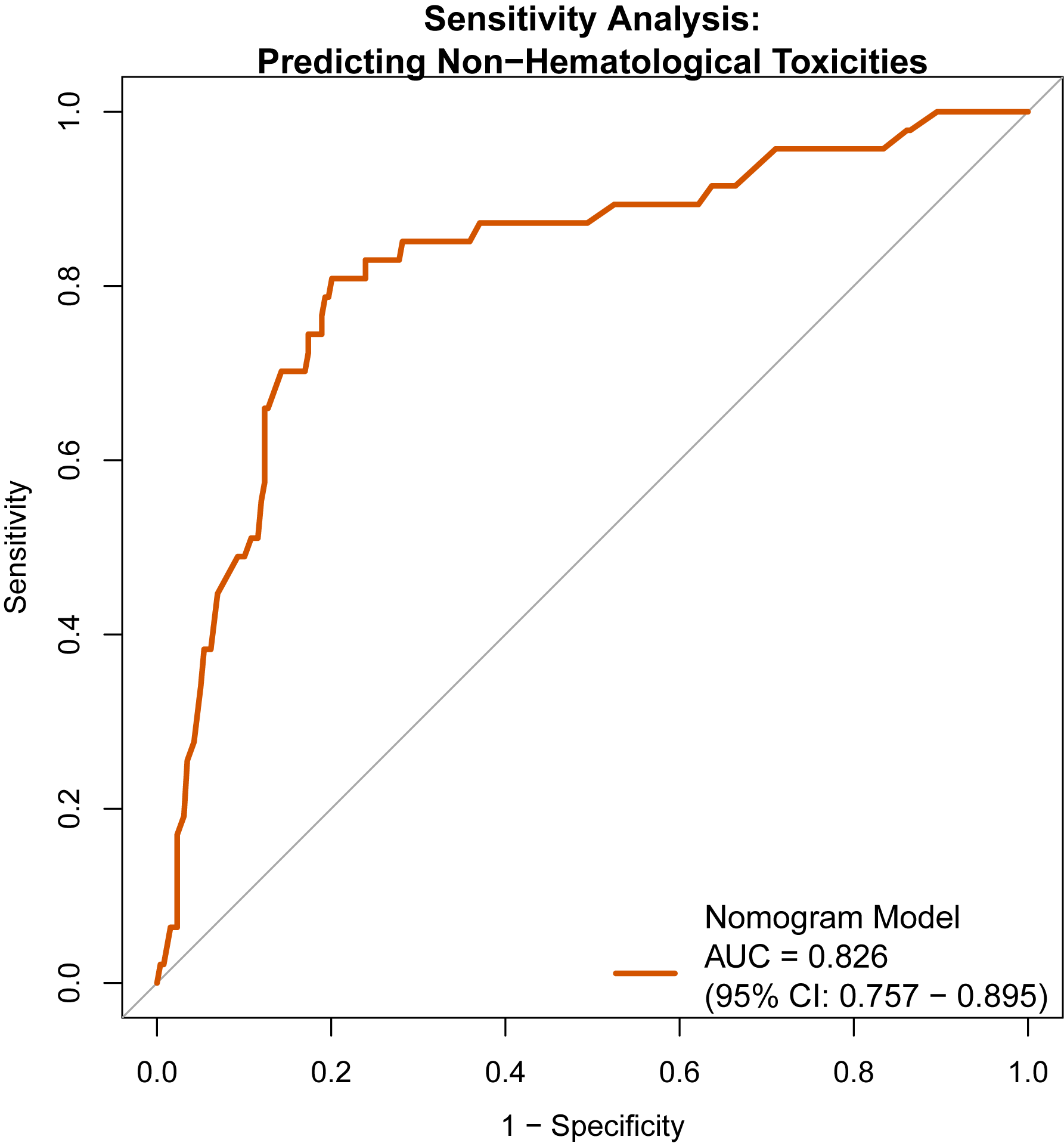

Supplement: Supplementary Figure 2 — Sensitivity analysis of the nomogram for predicting severe non-hematological toxicities. ROC curve evaluating the predictive performance of the composite model specifically for non-hematological adverse events (diarrhea, nausea/vomiting, liver dysfunction, hand-foot syndrome, and neurotoxicity), yielding an AUC of 0.826 (95% CI: 0.757–0.895). [file Image2.tif]
